# The Role of BMI and Blood Pressure in the Relationship Between Total Cholesterol and Disability in Chinese Centenarians: A Cross-Sectional Study

**DOI:** 10.3389/fmed.2021.608941

**Published:** 2021-02-16

**Authors:** Shengshu Wang, Wangping Jia, Shanshan Yang, Ke Han, Wenzhe Cao, Xueling Ren, Jing Li, Penggang Tai, Fuyin Kou, Miao Liu, Yao He

**Affiliations:** ^1^Institute of Geriatrics, Beijing Key Laboratory of Aging and Geriatrics, National Clinical Research Center for Geriatrics Diseases, Second Medical Center of Chinese PLA General Hospital, Beijing, China; ^2^Department of Disease Prevention and Control, The 1st Medical Center, Chinese PLA General Hospital, Beijing, China; ^3^Department of Respiratory, The 2nd Medical Center, Chinese People's Liberation Army General Hospital, Beijing, China; ^4^Medical Service Department, The 5th Medical Center, Chinese People's Liberation Army General Hospital, Beijing, China; ^5^Medical Service Department, Chinese People's Liberation Army General Hospital, Beijing, China; ^6^Department of Statistics and Epidemiology, Graduate School of Chinese PLA General Hospital, Beijing, China; ^7^State Key Laboratory of Kidney Diseases, Chinese People's Liberation Army General Hospital, Beijing, China

**Keywords:** total cholesterol, activities of daily living, disability, correlation, mediating effect

## Abstract

**Background:** Lower serum lipid metabolism might be associated with the decline of activity of daily living in the extreme longevity group. However, studies on models and possible paths of this correlation between total cholesterol (TC) and disability in centenarians are scarce. The aim of this study was to verify this correlation and explore the mediating effect of BMI and blood pressure on this relationship in Hainan centenarians.

**Methods:** We conducted a cross-sectional analysis of 1002 centenarians from the China Hainan Centenarians Cohort Study (CHCCS). Data on demographics, anthropometry data, lifestyle, and TC levels were collected through interviews, physical examinations, and laboratory tests. The Barthel index and Lawton index, measuring the disability status, were used to estimate the activity of daily living (ADL) and instrumental activity of daily living (IADL). A multivariable logistic regression model was used to explore the correlation between disability and TC levels. Mediation analyses were used to explore the both direct and indirect effects of TC level on disability.

**Results:** After adjusting for covariates, with 1 mmol/L increment in TC, the adjusted odds ratios (ORs) of ADL severe disability and ADL moderate & severe disability were 0.789(95%CI: 0.650–0.959) and 0.822(95%CI: 0. 0.699–0.966), respectively. There was a significant declining trend in the prevalence of different types of disability with increment in TC. The correlation was more pronounced among Hainan female centenarians. In the analysis of mediating effect among the female population, BMI significantly mediated the effect of TC levels on different types of disability. BMI and SBP, as chain mediators, multiply and chain mediated the effect of TC levels on IADL.

**Conclusion:** Low TC levels might be correlated with a higher frequency of disability in female centenarians, and this correlation might be mediated by BMI and blood pressure.

## Introduction

It is well-established that population aging is one of the significant challenges for many countries, especially for China (1). The sharp increase in older populations poses a major challenge to health and social security systems in that older people have a higher prevalence of disability (2–4). Activities of daily living (ADL) refers to necessary activities that people undertake routinely for meeting the needs of daily life, and it was usually used to estimate the decline or even loss of body function among older adults (5), while instrumental activity of daily living (IADL) is regarded as a significant indicator to assess the independence of living in society (6). Disability is often known as a problem affecting old people aged 65 or older and the effects of these problems usually increase with age. Considering the specificity of extreme longevity, ADL disability in the general old population cannot precisely represent the centenarians.

Hypercholesterolemia, a major causal risk factor for cardiovascular events, is considered as risk factors for longevity (7–9). However, in an older population, there is no consensus concerning the health impact of hypercholesterolemia on longevity and disability. Studies have shown that higher triglyceride may be related to better ADL among the older population (10, 11), and even higher lipid profiles are beneficial to longevity and functional performance among the older population (12–15). In addition, the influence pathway and physiological mechanism of the possible effect of blood lipids on disability were complex and uncertain. Moreover, no epidemiological evidence of the correlation and correlation ways of TC levels and disability, such as ADL and IADL, in centenarians.

The aims of the current study are to explore the possible correlation and potential correlated paths of TC level and disability in centenarians. We used data from the China Hainan Centenarians Cohort Study (CHCCS), which includes complete samples of community-based centenarians in Hainan, China.

## Materials and Methods

### Subjects

The data of this study were from the cross-sectional survey of CHCCS from 2014–2016. CHCCS is an ongoing whole-samples centenarians survey in Hainan, China, which was reported elsewhere (16). The first survey wave has been investigated from June 2014 to December 2016 *via* face-to-face investigation. It was carried out in a complete sample study, containing 1,811 living centenarians in 2014 according to the household register provided by the civil affairs bureau. We excluded 338 centenarians for whom we could not be reached based on the contact information provided. We also excluded individuals who were not conscious and could not perform the questionnaire interviews, physical health examination, and blood collection. A rigorous age validation process was conducted to prevent participants from exaggerating their age (16). Of 1,473 eligible individuals, a total of 1,002 centenarians aged 100 to 116 years, living in community, at enrollment in baseline survey were assessed for study eligibility. The study was approved by the Ethics Committee of the Chinese People's Liberation Army General Hospital (approval number: 301hn11201601). All centenarians signed the written informed consent forms.

### ADL Assessment

Barthel Index (5), and Lawton IADL Scale (17) were used to estimate whether it is a disability. Participants had been inquired if they could finish the items from the ADL scales. The Barthel ADL Index, containing 10 items with 0 points for inability to 10 or 15 points for complete independence, were summed to give a score from 0 to 100, and a total score of 0–20 suggested complete dependence, 21–60 severe dependence, 61–95 moderate dependence and 100 complete independence (18). The ADL questionnaire was answered by centenarians with health-conscious and checked by their relatives, and if they were incomplete health-conscious, the questionnaire was answered by their relatives (16). We defined ADL moderate and severe disability as reporting Barthel index score≤60, and ADL severe disability as reporting Barthel index score≤40 (19). The 8 items of Lawton IADL scale were summed to give a score from 0 to 8, and participants scored 8 were defined as IADL independent, 6–7 were defined as IADL mild disability, 3–5 were IADL moderate disability, and ≤2 were IADL severe disability (20).

### Measurement of TC and Other Covariates

The fasting blood was collected by experienced nurses using four vacutainer tubes (2 ml) to detect the total cholesterol level. Blood specimens were placed in a cold box and then were tested in the Laboratory of Hainan Branch of the Chinese PLA General Hospital within 6 h by automatic biochemical analyzer. To better assess the association of TC with ADL disability, TC level was analyzed both as continuous and categorical variables in quartiles, respectively. TC level was divided into quartile: Q1 (≤4.05 mmol/L), Q2 (4.06–4.60 mmol/L), Q3 (4.61–5.25), and Q4 (≥5.26mmol/L).

Information on age, gender, nationality, marital status, educational level, and residential type were obtained through the questionnaire. Centenarians' disease history and whether taking medicine were surveyed. As most centenarians with develop a hunchback, the height of the centenarians was measured as the length from the top of the head through the spine to the heel with a standard soft ruler. Body Mass Index(BMI) categorized as underweight (<18.5 kg/m^2^), normal weight (18.5–24.0 kg/m^2^), and overweight (≥24.0 kg/m^2^) (21). The blood pressure was measured twice in calm conditions by a portable electronic sphygmomanometer (1 mmHg = 0.133 kPa) at intervals of 1–2 min. Smoking status, alcohol use, and physical activity were investigated by self-reported. The physical activity was assessed by answering “how many times did you do physical activities related to independent life per week,” and we defined infrequent activity as once weekly or less (22). The Mini-mental State Examination (MMSE) scale was used to estimate the cognition status.

### Statistical Analyses

In this study, the Shapiro-Wilk test was used to estimate the normality of continuous variables and the results showed all continuous variables did not follow a normal distribution. Kruskal-Wallis test was used to test group differences, and the results were expressed as the median and interquartile range (IQR). The Chi-square test was used for intergroup comparison, and the results were expressed as number and percentage (*n*/%) since Barthel and Lawton scores following the non-normal distribution were analyzed as the categorical variables. The univariate and multivariable logistic regression models were used to explore the correlation between disability and TC levels. In multivariable analyses, demographic characteristics, health status, and physical activity were adjusted. We further excluded participants with dyslipidemia or taking lipid-lowering medicine to examine the possible correlation between disability and TC levels in sensitivity analyses. Odds Ratios (ORs) and 95% confidence interval (CI) were reported. SPSS24.0 was used for statistical analysis and EmpowerStats (http://www.empowerstats.com, X&Y Solutions, Inc., Boston, MA) and packages R (http://www.R-project.org, The R Foundation) for restrictive cubic spline function. All *P*-values were from two-sided tests, and *P* < 0.05 was used as the test level with statistical significance.

The possible mediations were explored according to the analytic methods outlined by Preacher and Hayes (23). All mediation analyses were carried out using PROCESS, which is an SPSS macro by Hayes (24, 25). The simple mediating effect was screened by PROCESS model 4, and the multiple chain mediated model by PROCESS model 6 (25). All mediating effects were based on 5,000 samples bootstrapping set and all estimated mediating effects reported in this study are unstandardized regression coefficients.

## Results

### Basic Characteristics of Centenarians

Among 1,002 centenarians, the median age was 102 years (interquartile range [IQR], 101–104 years), and 822 were women (82.0%). A total of 145 centenarians (14.5%) were identified as ADL severe disability and 648 centenarians (64.7%) as IADL severe disability (Table 1).

**Table 1:** Characteristics of total cholesterol level of hainan centenarians in China.

		**Quartile of TC Levels (mmol/L)**				
	**Total**	**Q1 (≤4.05)**	**Q2 (4.06–4.60)**	**Q3 (4.61–5.25)**	**Q4 (≥5.26)**	***P*-value**
	**(*n* = 1,002)**	**(*n* = 252)**	**(*n* = 279)**	**(*n* = 227)**	**(*n* = 244)**	
Ages, median (IQR), y	102.0 (101.0–104.0)	102 (101–104)	102 (101–104)	102 (101–104)	102 (101–104)	0.902
BMI, median (IQR), kg/m^2^	17.9 (16.0–19.9)	17.6 (15.6–19.2)	17.9 (16.2–9.9)	18.4 (16.3–20.4)	18.2 (16.4–20.1)	0.028
SBP, median (IQR), mmHg	152.0 (136.5–166.82)	146.4 (130.1–163.0)	138 (152.4–166.8)	150.8 (137–168)	152.4 (140.3–169.9)	0.009
DBP, median (IQR), mmHg	75.7 (67.5–81.5)	73.4 (64.0–79.4)	67.5 (75.7–81.8)	75 (68–79.8)	76 (70.5–84)	0.003
TG, median (IQR), mmol/L	1.1 (0.8–1.38)	0.87 (0.66–1.11)	0.8 (1–1.3)	1.1 (0.9–1.5)	1.2 (0.9–1.6)	<0.001
LDL-C median (IQR), mmol/L	2.8 (2.3–3.2)	2.0 (1.7–2.3)	2.4 (2.6–2.8)	3 (2.7–3.2)	3.7 (3.3–4.1)	<0.001
HDL-C median (IQR), mmol/L	1.4 (1.2–1.7)	1.2 (1.0–1.4)	1.2 (1.4–1.6)	1.5 (1.2–1.8)	1.6 (1.3–1.9)	<0.001
FPG median (IQR), mmol/L	5.0 (4.3–5.6)	5.0 (4.2–5.9)	4.3 (5–5.5)	4.9 (4.2–5.7)	4.9 (4.3–5.6)	0.555
**Gender**						<0.001
Male	180 (17.96)	81 (26.05)	40 (14.6)	33 (14.73)	26 (13.47)	
Female	822 (82.04)	230 (73.95)	234 (85.4)	191 (85.27)	167 (86.53)	
**BMI classification**						0.007
<18.5 kg/m^2^	575 (57.39)	190 (61.09)	163 (59.49)	114 (50.89)	108 (55.96)	
18.5–24.0 kg/m^2^	390 (38.92)	118 (37.94)	102 (37.23)	94 (41.96)	76 (39.38)	
≥24.0kg/m^2^	37 (3.69)	3 (0.96)	9 (3.28)	16 (7.14)	9 (4.66)	
**Nationality**						0.062
Han	883 (88.12)	216 (85.71)	240 (86.02)	201 (88.55)	226 (92.62)	
Ethnic minority	119 (11.88)	36 (14.29)	39 (13.98)	26 (11.45)	18 (7.38)	
**Educational level**						0.976
Illiteracy	915 (91.32)	230 (91.27)	254 (91.04)	208 (91.63)	223 (91.39)	
Primary school	67 (6.69)	18 (7.14)	20 (7.17)	13 (5.73)	16 (6.56)	
Junior high school	20 (2)	4 (1.59)	5 (1.79)	6 (2.64)	5 (2.05)	
and above						
**Marital status**						0.868
Married	100 (9.98)	28 (11.11)	25 (8.96)	22 (9.69)	25 (10.25)	
Widowed/divorced/	902 (90.02)	224 (88.89)	254 (91.04)	205 (90.31)	219 (89.75)	
unmarried						
**Residential type**						0.676
Living with family	863 (86.13)	221 (87.7)	242 (86.74)	195 (85.9)	205 (84.02)	
Living alone/nursing	139 (13.87)	31 (12.3)	37 (13.26)	32 (14.1)	39 (15.98)	
home						
**ADL**						0.023
Independent	165 (16.5)	37 (22.4)	40 (24.2)	40 (24.2)	48 (29.1)	
Mild disability	550 (54.9)	123 (22.4)	154 (28)	136 (24.7)	137 (24.9)	
Moderate disability	142 (14.2)	40 (28.2)	46 (32.4)	28 (19.7)	28 (19.7)	
Severe disability	145 (14.5)	52 (35.9)	39 (26.9)	23 (15.9)	31 (21.4)	
**IADL**						0.006
Independent/Mild/	354 (35.3)	67 (18.9)	102 (28.8)	85 (24)	100 (28.2)	
Moderate disability						
Severe disability	648 (64.7)	185 (28.5)	177 (27.3)	142 (21.9)	144 (22.2)	

*ADL, activities of daily living; IQR, interquartile range; BMI, body mass index; SBP, Systolic blood pressure; DBP, diastolic blood pressure; TG, triglyceride; FPG, fasting plasma glucose, LDL-C, low-density lipoprotein cholesterol; HDL-C, high-density lipoprotein cholesterol*.

### Distribution of TC Level and ADL Scale

**Figure 1:**
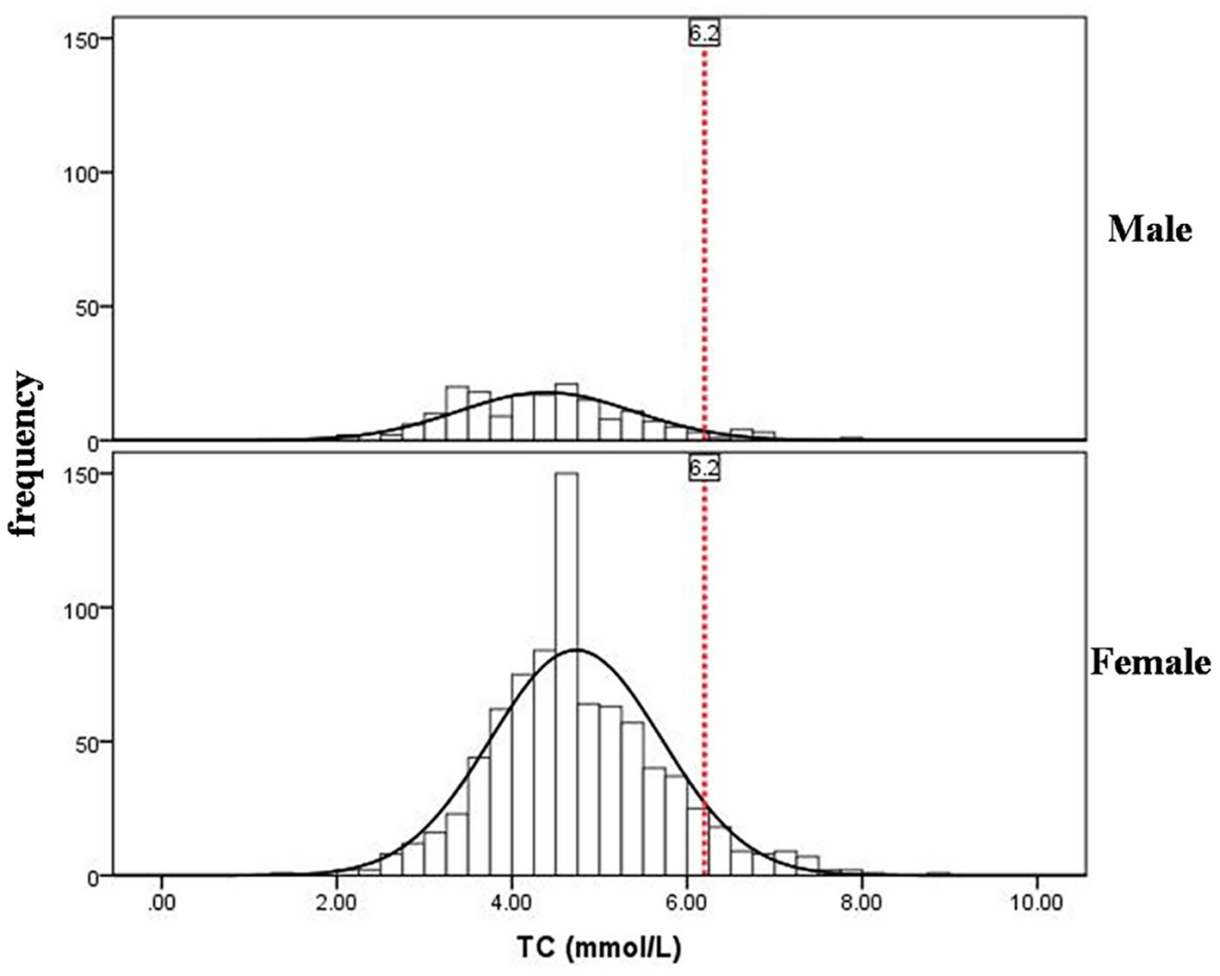
Distribution of serum total cholesterol levels in male and female centenarians. The red dotted lines represent normal reference values.

**Figure 2:**
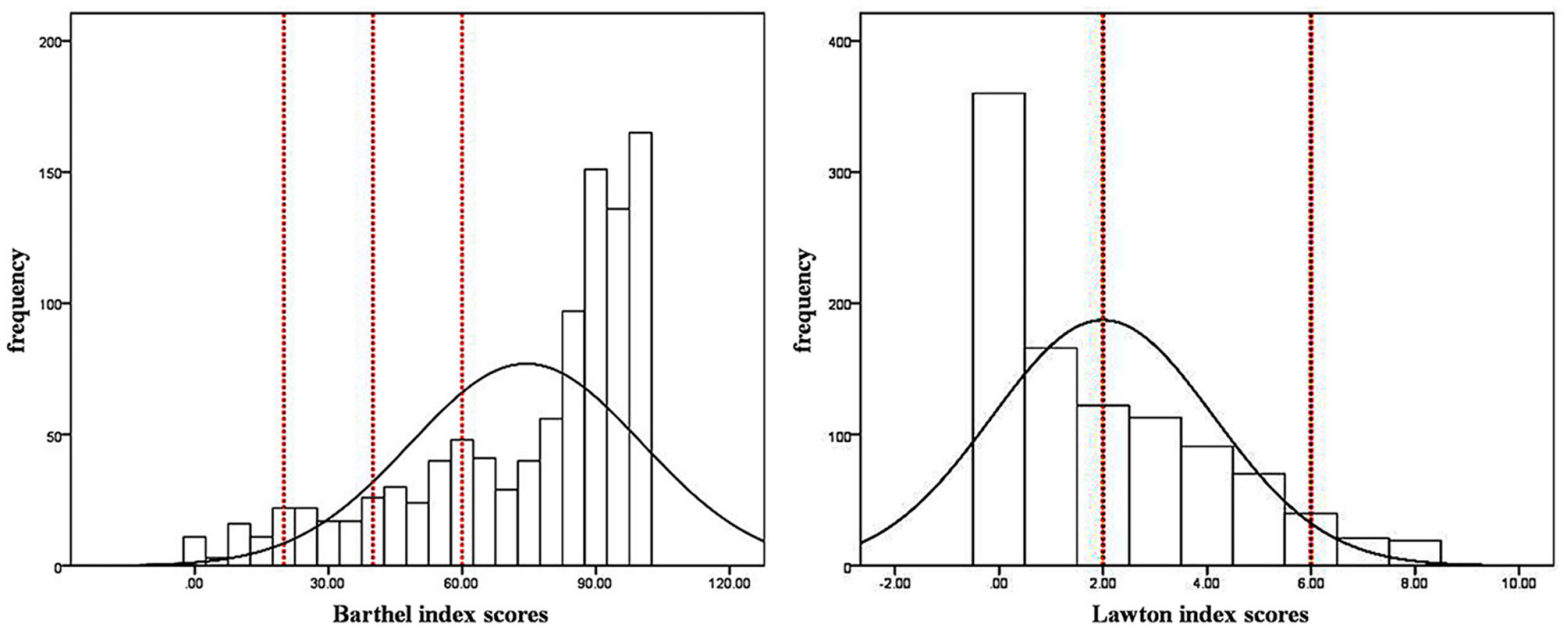
Distribution of Barthel index scores and Lawton index scores in Hainan centenarians. The red dotted lines represent the cut-off value of different degrees of disability.

Histogram showed the sex differences in the distribution of serum TC levels were significant and female centenarians had a higher level of TC (Figure 1). The TC median value in female centenarians (4.60 mmol/L) was higher than the median value in males (4.35 mmol/L). Histograms showed the distributions of Barthel index and Lawton index were significantly skewed distribution (Figure 2).

### The Correlations Between TC Levels and Disability

**Figure 3:**
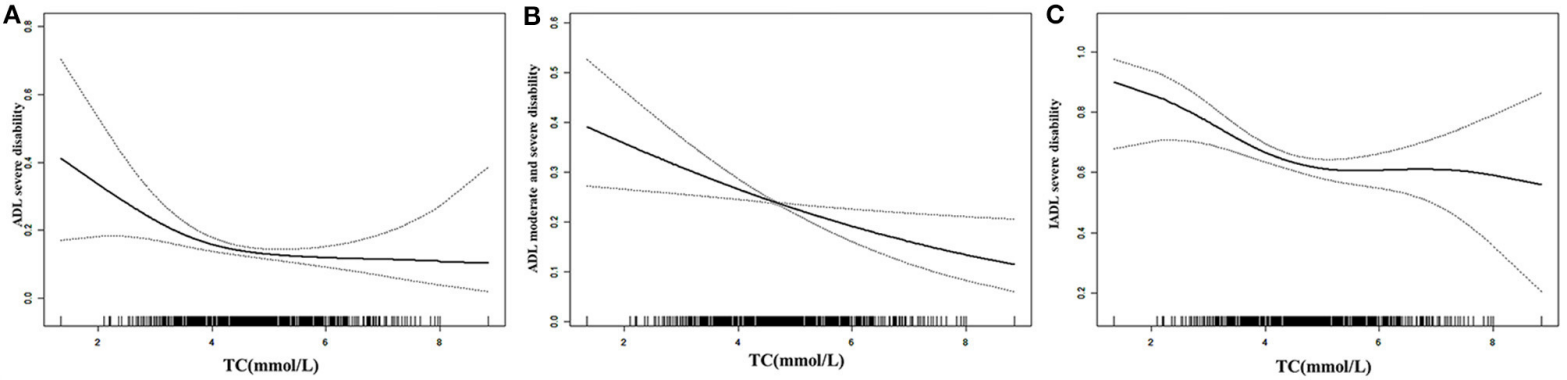
Cubic spline curve of the inverse correlation between Total Cholesterol Levels and different types of disability [**(A)**, ADL moderate and severe disability; **(B)**, ADL severe disability; **(C)**, IADL severe disability] in Hainan centenarians after adjusting for covariates.

The results of the multivariate Logistic regression model showed that, in the female centenarian population, after adjusting fully for potential confounding factors, the risk of ADL severe disability, ADL moderate and severe disability decreased by 21.1% (OR = 0.789, 95%CI:0.650–0.959) and 27.8% (OR = 0.822, 95%CI:0.699–0.966), respectively, with the increment of 1 mmol / L of TC level. No significant correlation was found in male centenarians (Table 2 and Figure 3).

**Table 2:** The odds ratio of TC levels for disability in the multivariate logistic regression model.

	**ADL severe disability**	**ADL moderate and severe disability**	**IADL severe disability**
**ALL CENTENARIANS (*****n*** **=** **1,002)**			
Crude model	**0.767(0.636–0.925)**	**0.784(0.672–0.914)**	**0.819(0.718–0.934)**
Model 1	**0.746(0.615–0.904)**	**0.760(0.649–0.889)**	**0.807(0.703–0.926)**
Model 2	**0.746(0.615–0.904)**	**0.764(0.652–0.895)**	**0.831(0.722–0.955)**
Model 3	**0.789(0.650–0.959)**	**0.822(0.699–0.966)**	0.867(0.746–1.007)
**MALE (*****n*** **=** **180)**			
Crude model	0.983(0.603–1.601)	0.880(0.595–1.301)	**0.676(0.497–0.920)**
Model 1	0.845(0.490–1.460)	0.886(0.597–1.314)	0.729(0.531–1.002)
Model 2	0.833(0.476–1.458)	0.891(0.589–1.348)	0.753(0.539–1.050)
Model 3	0.905(0.497–1.649)	0.891(0.553–1.436)	0.754(0.513–1.108)
**FEMALE (*****n*** **=** **822)**			
Crude model	**0.707(0.574–0.870)**	**0.740(0.623–0.878)**	**0.814(0.700–0.945)**
Model 1	**0.724(0.587–0.893)**	**0.740(0.623–0.878)**	**0.830(0.711–0.968)**
Model 2	**0.728(0.589–0.900)**	**0.741(0.624–0.881)**	**0.850(0.727–0.994)**
Model 3	**0.746(0.602–0.924)**	**0.781(0.652–0.934)**	0.883(0.746–1.044)

*Model 1: Adjusted for age, gender, BMI, nationality, marital status, educational level, and residential type*.

*Model 2: Adjusted for age, gender, BMI, nationality, marital status, educational level, residential type, glucose, and DBP*.

*Model 3: Adjusted for age, gender, BMI, nationality, marital status, educational level, residential type, glucose, DBP, MMSE, smoking, drinking, and physical activity. Bold indicate statistically significant OR and 95% confidence interval*.

### The Correlations Between TC Quartile and Disability

After adjustment, compared to centenarians with TC 1st quartile, the risk of TC 4th quartile of ADL moderate and severe disability and IADL severe disability decreased by 46.1% (OR = 0.639, 95%CI: 0.415–0.984), and 36.4% (OR = 0. 636, 95%CI: 0.414–0.977), respectively (Table 3).

**Table 3:** The odds ratio (ORs) of TC categorical variables for ADL severe disability and ADL moderate and severe disability in the multivariate logistic regression model, compared the Q1.

	**Quartile of TC Levels (mmol/L)**				
	**Q1 (≤4.05) (*n* = 252)**	**Q2 (4.06–4.60) (*n* = 279)**	**Q3 (4.61–5.25) (*n* = 227)**	**Q4 (≥5.26) (*n* = 244)**	**P for trend**
**ADL SEVERE DISABILITY**					
Crude model	1	**0.625 (0.396–0.986)**	**0.434 (0.256–0.735)**	**0.560 (0.345–0.909)**	0.006
Model 1	1	**0.594 (0.374–0.942)**	**0.403 (0.236–0.688)**	**0.532 (0.325–0.870)**	0.006
Model 2	1	**0.608 (0.382–0.968)**	**0.415 (0.242–0.711)**	**0.549 (0.332–0.907)**	0.006
Model 3	1	**0.665 (0.411–1.045)**	**0.459 (0.268–0.787)**	0.616 (0.373–1.019)	0.021
**ADL MODERATE AND SEVERE DISABILITY**					
Crude model	1	0.762 (0.531–1.094)	**0.504 (0.337–0.755)**	**0.555 (0.376–0.819)**	0.001
Model 1	1	0.765 (0.531–1.100)	**0.506 (0.338–0.760)**	**0.565 (0.382–0.836)**	0.001
Model 2	1	0.757 (0.523–1.096)	**0.500 (0.331–0.757)**	**0.568 (0.380–0.851)**	0.001
Model 3	1	0.782 (0.526–1.163)	**0.544 (0.349–0.847)**	**0.639 (0.415–0.984)**	0.040
**IADL SEVERE DISABILITY**					
Crude model	1	**0.628 (0.434–0.911)**	**0.605 (0.411–0.892)**	**0.522 (0.357–0.761)**	0.001
Model 1	1	**0.599 (0.407–0.881)**	**0.576 (0.383–0.866)**	**0.503 (0.338–0.749)**	0.003
Model 2	1	**0.620 (0.420–0.914)**	**0.600 (0.398–0.905)**	**0.549 (0.367–0.822)**	0.006
Model 3	1	0.679 (0.448–1.030)	0.664 (0.429–1.028)	**0.636 (0.414–0.977)**	0.009

*Model 1: Adjusted for age, BMI, nationality, marital status, educational level, and residential type*.

*Model 2: Adjusted for age, BMI, nationality, marital status, educational level, residential type, glucose, and DBP*.

*Model 3: Adjusted for age, BMI, nationality, marital status, educational level, residential type, glucose, DBP, MMSE, smoking, drinking, and physical activity. Bold indicate statistically significant OR and 95% confidence interval*.

### Sensitivity Analysis

We also analyzed the association of TC levels with disability among 710 female centenarians without dyslipidemia who were taking lipid-lowering medicine. After adjustment, the risk of ADL severe disability, ADL moderate and severe disability, and IADL severe disability decreased with the increment of TC indicators (Table 4).

**Table 4:** Correlations of TC indicators and disability among female Hainan 435 centenarians without dyslipidemia, previous hyperlipidemia, and taking lipid-lowering medicine^a^.

	**TC Level**	**TC Quartile of Female Centenarians (*****n*** **=** **736)**				
		**Q1 (<4.07) (*n* = 185)**	**Q2 (4.08–4.59) (*n* = 185)**	**Q3 (4.60–5.11) (*n* = 184)**	**Q4 (>5.12) (*n* = 182)**	**P for Trend**
**ADL SEVERE DISABILITY**						
Unadjusted	0.654 (0.507–0.842)	1	0.334 (0.183–0.612)	0.593 (0.350–1.004)	0.526 (0.306–0.905)	0.001
Adjusted^b^		1	0.345 (0.183–0.652)	0.600 (0.341–1.056)	0.554 (0.308–0.996)	0.107
**ADL MODERATE/SEVERE DISABILITY**						
Unadjusted	0.680 (0.549–0.842)	1	0.538 (0.340–0.853)	0.577 (0.365–0.910)	0.482 (0.301–0.773)	<0.001
Adjusted^b^		1	0.560 (0.338–0.930)	0.577 (0.351–0.950)	0.493 (0.292–0.831)	0.010
**IADL SEVERE DISABILITY**						
Unadjusted	0.787 (0.644–0.963)	1	0.580 (0.369–0.910)	0.603 (0.383–0.948)	0.639 (0.404–1.006)	0.020
Adjusted^b^		1	0.642 (0.375–1.101)	0.609 (0.358–1.038)	0.751 (0.434–1.299)	0.317

a *Results weighted to sensitivity analysis estimates which have been balanced the complex confounding factors*.

b *All models adjusted for age, BMI, nationality, marital status, educational level, residential type, glucose, DBP, MMSE, smoking, drinking, and physical activity*.

### The Mediation Analysis

**Figure 4:**
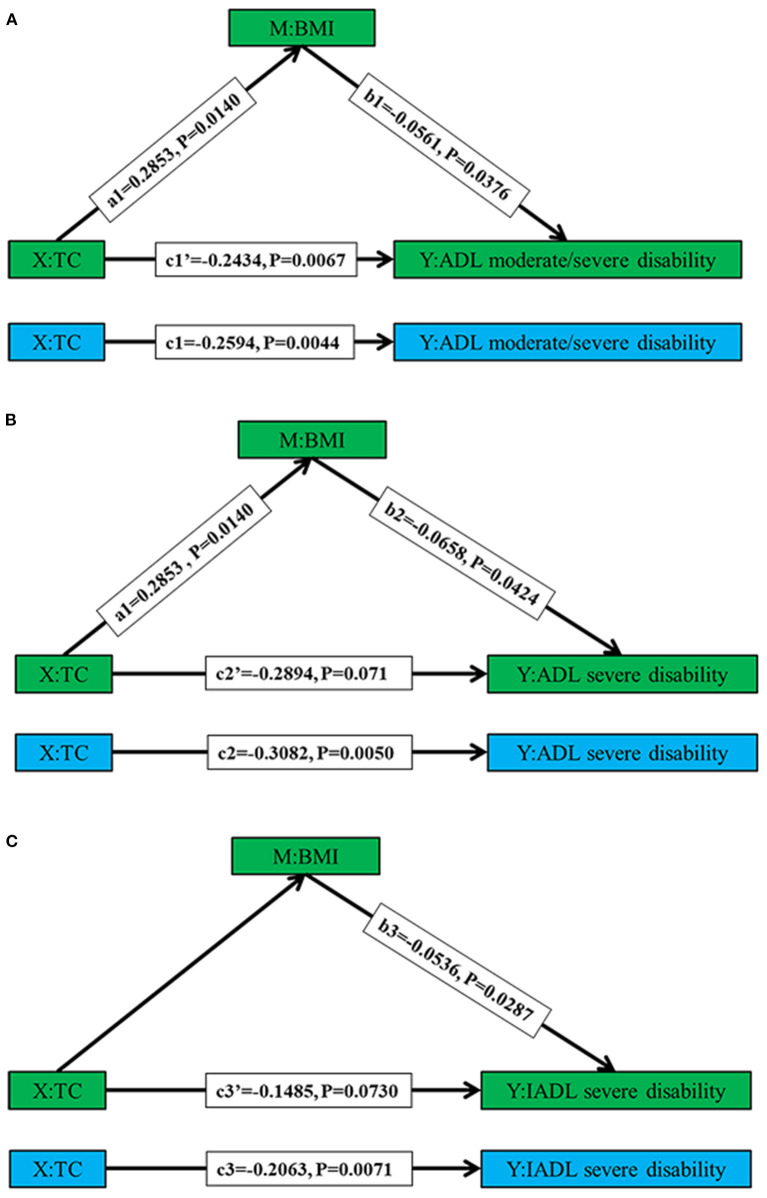
The simple mediating effects of BMI between TC levels (X) and different types of disability (Y). The blue graphs depicting the total effect (c) of TC on different types of disability (Y), and the green graphs depicting the direct effect (c') and mediating effects of BMI on TC on different types of disability (Y). Graph **A–C** representing different types of disability (ADL moderate/severe disability, ADL severe disability, and IADL severe disability), respectively. Effect values refer to unstandardized regression coefficients.

We examined, among Chinese female centenarians, whether BMI and blood pressure indicators (SBP and DBP) mediated the relationship between TC levels and different types of disability (ADL moderate/severe disability, ADL severe disability, and IADL severe disability), respectively, adjusting for the age, nationality, educational level, marital status, residential type, FPG, MMSE, smoking, and drinking. The results of mediating effect indicated lower serum lipid, lower blood pressure, and lower BMI may be correlated with functional impairment in female centenarians, and simple mediating effect showed BMI mediating the effect of TC levels on different types of disability (ADL moderate/severe disability, ADL severe disability and IADL severe disability) was significant, accounted for 6.17, 6.10, and 7.42% of the total effect, respectively (Table 5 and Figure 4).

**Table 5:** The mediating effect of BMI between TC levels and different types of disability.

	**Coefficient**	**Standard deviation**	**t-value**	***P***	**Total effect**	**Mediating effect**
ADL moderate/severe disability					−0.2594	−0.0160
TC→disability	−0.2594	0.0981	−2.7488	0.0044		
TC→BMI	0.2853	0.1159	2.4615	0.0140		
BMI→disability	−0.0561	0.0270	−2.0792	0.0376		
TC'→disability	−0.2434	0.0897	−2.7131	0.0067		
ADL severe disability					−0.3082	−0.0188
TC→disability	−0.3082	0.1200	−2.5198	0.0050		
TC→BMI	0.2853	0.1159	2.4615	0.0140		
BMI→disability	−0.0658	0.0324	−2.0297	0.0424		
TC'→disability	−0.2894	0.1076	−2.6901	0.0071		
IADL severe disability					−0.2063	−0.0153
TC→disability	−0.2063	0.0764	−2.6995	0.0071		
TC→BMI	0.2853	0.1159	2.4615	0.0140		
BMI→disability	−0.0536	0.0245	−2.1872	0.0287		
TC'→disability	−0.1485	0.0828	−1.7928	0.0730		

**Figure 5:**
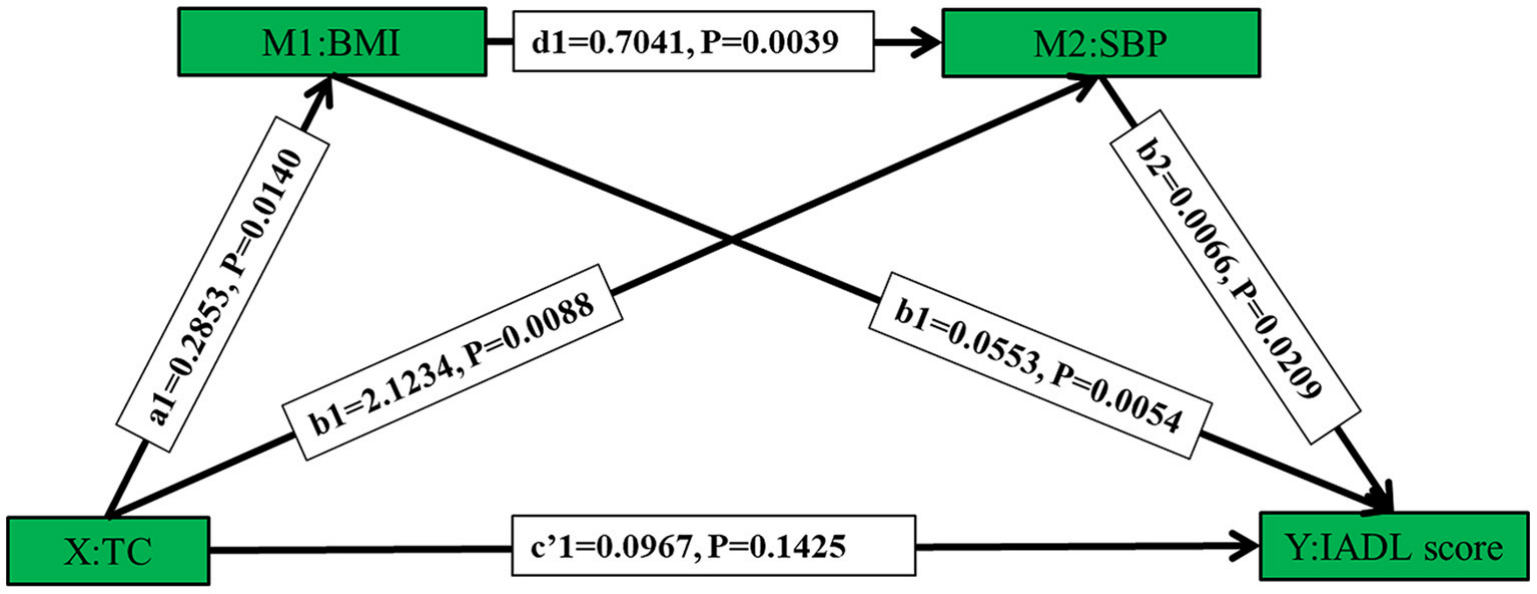
The multiple-chain-mediated model, including multiple mediating effect and chain mediating effects, depicting direct and indirect effects of TC levels (X) on IADL scores (Y). BMI and SBP, as a mediator as well as chain mediators, partially mediate the effect of total cholesterol on disability. Effect values refer to unstandardized regression coefficients.

After adjusting for covariates, the results of the multiple chain mediated model showed a significant chain mediating effect through the path completely accomplished by chain mediators BMI and SBP (B = 0.0013, *P* = 0.0010), and two significant simple mediating paths mediated by BMI (B = 0.0158, *P* = 0.0131), and SBP (B = 0.0139, *P* = 0.0085), respectively. The effect of TC on IADL was completely mediated by BMI and SBP (Table 6 and Figure 5).

**Table 6:** The effect values of mediating paths from TC to IADL in the multiple chain mediated model among female centenarians.

**Paths**	**Coefficient**	**95%CI**	***P***
TC→BMI	0.2853	0.0578–0.5128	0.0140
TC→SBP	2.1234	0.5354–3.7114	0.0088
BMI→IADL scores	0.0553	0.0164–0.0941	0.0054
SBP→IADL scores	0.0066	0.0010–0.0121	0.0209
BMI→SBP	0.7041	0.2271–1.1811	0.0039
TC→IADL scores	0.0967	−0.0326–0.2259	0.1425
TC→BMI→IADL scores	0.0158	0.0022–0.0357	0.0131
TC→BMI→SBP→IADL scores	0.0013	0.0001–0.0037	0.0010
TC→SBP→IADL scores	0.0139	0.0010–0.0329	0.0085

*The effect value of mediating paths of TC→BMI→IADL scores is the product of TC→BMI and BMI→IADL scores; The effect value of mediating paths of TC→SBP→IADL scores is the product of TC→SBP and SBP→IADL scores; The effect value of mediating paths of TC→BMI→SBP→IADL scores is the product of TC→BMI, BMI→SBP and BMI→IADL scores*.

## Discussion

The main finding from this cross-sectional and complete samples study on Hainan centenarians suggest that elevated TC was inversely correlated with disability, and the association might be mediated by BMI and SBP in the female centenarian population. To our knowledge, this is the first study describing the epidemiological correlation between TC level and disability, and to quantify the mediating effect of this relationship among a complete sample female centenarian population.

Similar to other studies, our study found that, in Hainan centenarians, the prevalence of disability was high and the TC levels were moderate (26, 27). The variability among different studies might be attributed to different ADL scales. Several studies have confirmed that the TC level in centenarians was generally within the normal range, and was lower than that in the healthy older adults control group (28, 29).

In this study, the elevated TC levels in female Hainan centenarians were correlated with disability among centenarians. This possible relationship was still stable in sensitivity analyses. However, the association we deduced was inconsistent with those concluded among the different age groups. A 32-year follow-up survey from Chicago Heart Association Detection Project suggested that lower TC levels were associated with the lowest rate of functional disability in older age (30). Among the younger- and older-old from Sweden, the results suggested there was no statistical correlation between blood lipids and ADL disability (31). Lower total cholesterol and high-density lipoprotein cholesterol levels were associated with a higher risk of ADL disability (32, 33).

Inconsistent correlations might be partially attributable to age difference in that the old with higher serum lipid levels were more likely to die early from cardiovascular diseases, and it might be also explained that centenarians might have unique physiological reference values of blood lipids profile, which was significantly different from that of younger adults, but the current physiological reference standards for blood lipid profile was not standardized according to age (34). The inverse correlation between elevated TC level and disability was only found in female centenarians. Gender differences may be mainly caused by different physiological characteristics (34).

The previous studies have shown that older adults' functional impairment might be related to malnutrition (35, 36). In the current study, TC and BMI levels can represent some nutritional status of the centenarians. Lipids profile is influenced by comprehensive, whole-life cycle heredity, diet, and nutrition. BMI can reflect the degree of nutrition and obesity. 57.39% of Hainan centenarians were underweight, and 7% were hypercholesterolemia. Centenarians have a thinner body shape and lower TC levels than those adults. BMI was positively correlated to serum the TC level. It was suggested that the possible correlation between the BMI and TC levels may be significant, both as indicators for assessing the nutrition status. Therefore, in this study, BMI was considered as a confounding factor to be adjusted in the multivariate logistic regression model and treated as a possible mediator in mediating effect analysis.

The results of the simple mediating effect suggested that BMI mediated the influence of TC on disabilities, which meant the higher the TC level, the higher the BMI, and the latter was related to less likely the risk of disability. The effects of TC on ADL disabilities were partially mediated by BMI. The results of the multiple-chain-mediating effect suggested BMI and SBP completely mediated this association either singly or in combination, which indicated the level of total cholesterol might indirectly and variously affect the disability through BMI and SBP. At present, no similar studies have been found and the causality deserves further verification. Nevertheless, some interesting perspectives for future research were presented. The potential public health meanings were that serum lipid, BMI, and blood pressure should be further noticed among the extreme longevity.

Although the exact mechanism remains unclear, several plausible mechanisms may underlie the inverse correlation between TC levels and ADL disability. First, cholesterol is involved in many important biochemical pathways (37), which may indirectly affect the ADLs of centenarians (38). Secondly, inflammatory markers associated with low total cholesterol levels were found to be associated with disability and poor Barthel ADL scores in older people (39). Cholesterol and its metabolites may regulate interleukin-6 signaling (40). Thirdly, cholesterol plays an important role in the formation and deposition of synapses, membranes, and amyloid-β, which affect brain function (41).

Our findings, similar to other studies, found the gender differences between TC and disability in centenarians. The results from the Chinese Longitudinal Healthy Longevity Survey (CLHLS) indicated that higher levels of total cholesterol and triglyceride within the normal range had protective effects on functional impairment of older people and particularly females (42, 43). We postulated that inversely associations of TC levels and ADL disability among female centenarians are as follows: (1) it is believed that women live longer in extremely long-lived populations, but men exhibit higher levels of cognitive function and ADL (44–46); (2) survivor bias, women were more likely to experience the higher prevalence of disability, was hard to rule out.

This study has some limitations. The conclusions that emerged from this cross-sectional study are not the real causal links between TC level and disability among centenarians but raise several intriguing speculations in such an extreme longevity population for further identification and research. Second, along with the Chinese nationality, there are possible associations of this study that are less generalizable to other regions and populations. Third, the results of the Barthel index and Lawton scale were self-reported. Bias would be difficult to avoid and was likely to occur. Fourth, due to the lower prevalence of hypercholesterolemia in centenarians, TC level was quartered to better describe its distribution characteristics, which was biased that the results of this quartering method only represented this centenarian population and could not explain the health impact of hypercholesterolemia on ADLs.

## Conclusion

This study is the first to demonstrate that low TC levels might be correlated with a higher frequency of disability in female centenarians, and this correlation might be mediated by BMI and blood pressure either singly or in combination. Accordingly, we hypothesize that lower serum lipid, blood pressure, and BMI may be detrimental to function in centenarians. Significantly, our positive results emerged from a cross-sectional design that could not draw any causal conclusions, and even the inversion of cause and effect cannot be ruled out. But these possible links, between disability and its related serum lipid indicators, may provide a way of thinking and hypothesis to be explored and identified.

## Data Availability Statement

The datasets China Hainan Centenarians Cohort Study (CHCCS) for this study was available upon request.

## Ethics Statement

The studies involving human participants were reviewed and approved by the Ethics Committee of Chinese People's Liberation Army General Hospital (approval number: 301hn11201601). The patients/participants provided their written informed consent to participate in this study.

## Author Contributions

SW, WJ, and SY were involved in the conception and design of the work. SW and WJ contributed to writing the manuscript. KH, WC, and XR contributed to data arrangement and statistical analysis. SY, WC, and WJ contributed to the data compilation. ML, XR, and YH contributed to critical review. PT, FK, and JL contributed to the literature search. SY, WJ, and SW contributed to the design of tables and figures. All authors contributed to the article and approved the submitted version.

## Conflict of Interest

The authors declare that the research was conducted in the absence of any commercial or financial relationships that could be construed as a potential conflict of interest.
